# Extracellular Signal-Regulated Kinase Is a Direct Target of the Anti-Inflammatory Compound Amentoflavone Derived from *Torreya nucifera*

**DOI:** 10.1155/2013/761506

**Published:** 2013-07-21

**Authors:** Jueun Oh, Ho Sik Rho, Yanyan Yang, Ju Young Yoon, Jongsung Lee, Yong Deog Hong, Hyeon Chung Kim, Sun Shim Choi, Tae Woong Kim, Song Seok Shin, Jae Youl Cho

**Affiliations:** ^1^Department of Genetic Engineering, Sungkyunkwan University, Suwon 440-746, Republic of Korea; ^2^Medical Beauty Research Institute, AmorePacific R&D Center, Yongin 446-729, Republic of Korea; ^3^Department of Dermatological Health Management, College of Health Science, Eulji University, Seongnam 461-713, Republic of Korea; ^4^College of Biomedical Sciences, Kangwon National University, Chuncheon 200-701, Republic of Korea; ^5^College of Natural Sciences, Kangwon National University, Chuncheon 200-701, Republic of Korea

## Abstract

Amentoflavone is a biflavonoid compound with antioxidant, anticancer, antibacterial, antiviral, anti-inflammatory, and UV-blocking activities that can be isolated from *Torreya nucifera, Biophytum sensitivum*, and *Selaginella tamariscina*. In this study, the molecular mechanism underlying amentoflavone's anti-inflammatory activity was investigated. Amentoflavone dose dependently suppressed the production of nitric oxide (NO) and prostaglandin E_2_ (PGE_2_) in RAW264.7 cells stimulated with the TLR4 ligand lipopolysaccharide (LPS; derived from Gram-negative bacteria). Amentoflavone suppressed the nuclear translocation of c-Fos, a subunit of activator protein (AP)-1, at 60 min after LPS stimulation and inhibited the activity of purified and immunoprecipitated extracellular signal-regulated kinase (ERK), which mediates c-Fos translocation. In agreement with these results, amentoflavone also suppressed the formation of a molecular complex including ERK and c-Fos. Therefore, our data strongly suggest that amentoflavone's immunopharmacological activities are due to its direct effect on ERK.

## 1. Introduction

Inflammation is a natural defense mechanism that protects the human body from various infections [1]. Inflammatory responses include the production of cytokines, such as interleukin (IL)-1, IL-6, IL-8, and tumor necrosis factor (TNF)-*α*; chemokines, such as monocyte chemotactic protein (MCP)-1; and inflammatory mediators, such as nitric oxide (NO) and prostaglandin E_2_ (PGE_2_), by various inflammatory cells, including epithelial cells, macrophages, keratinocytes, mast cells, and Langerhans cells [2, 3]. Molecularly, inflammation involves numerous intracellular signaling cascades, including the Src and Syk nonreceptor protein tyrosine kinases, phosphoinositide 3-kinase (PI3 K), phosphoinositide-dependent kinase 1 (PDK1), and Akt (protein kinase B) serine-threonine protein kinases, which contribute to the activation and upregulation of the transcription factors nuclear factor (NF)-*κ*B and activator protein (AP)-1 [4, 5]. Although inflammation is critical for maintaining health in the face of infection, exaggerated immune responses can cause serious diseases, such as cancer, atherosclerosis, and diabetes [6, 7].

**Figure 1:**
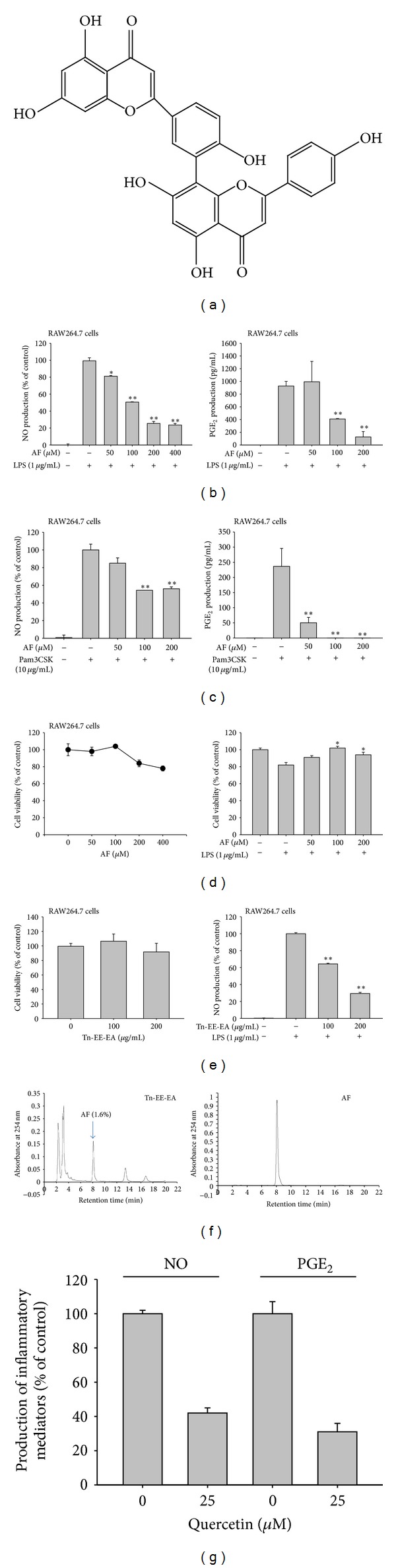
Effect of amentoflavone and Tn-EE-EA on the production of inflammatory mediators. (a) Chemical structure of amentoflavone (AF). (b, c, and g) Production of NO and PGE_2_ in RAW264.7 cells treated with LPS (1 *μ*g/mL) or pam3CSK (10 *μ*g/mL) in the presence or absence of amentoflavone (from 0 to 200 *μ*M) or quercetin (25 *μ*M) was measured using the Griess assay and EIA. ((d) and (e), right panel) The viability of RAW264.7 cell treated with amentoflavone (from 0 to 400 *μ*M) or Tn-EE-EA (0 to 200 *μ*g/mL) in the presence or absence of LPS was determined using an MTT assay. (e) Production of NO in RAW264.7 cells treated with LPS (1 *μ*g/mL) in the presence or absence of Tn-EE-EA (from 0 to 200 *μ*g/mL) was measured using the Griess assay (left panel). (f) The content of amentoflavone from Tn-EE-EA was analysed using a high-performance liquid chromatograph (HPLC) (Knauer). *P < 0.05 and **P < 0.01 compared to the control.

Amentoflavone (AF, Figure 1(a)) is a biflavonoid compound isolated from plants such as *Torreya nuncifera* Siebold et Zuccarini (Taxaceae), *Biophytum sensitivum*, *Origanum majorana*, *Cnestis ferruginea*, *Calophyllum flavoramulum*, *Byrsonima crassa*, and *Selaginella tamariscina *[8–10]. Like other biflavonoids, this compound has been reported to possess many biological activities, including antioxidant, anticancer, antibacterial, antiviral, anti-inflammatory, and UV-blocking effects [11–13]. The molecular mechanisms underlying these disparate effects are unknown. Although several pharmacological targets of amentoflavone (such as fatty acid synthase and suppressor of cytokine signaling 3 (SOCS3)) have been described [14, 15], these targets are not enough to explain the multiple activities of amentoflavone.

Recent studies have shown that amentoflavone treatment can decrease the production of cytokines such as tumor necrosis factor- (TNF-) *α* and inflammatory mediators such as nitric oxide (NO) and arachidonate by tumor-associated macrophages, peritoneal macrophages, and RAW264.7 cells [16, 17]. Though the effect of amentoflavone is very clear, its mechanism of action is not yet fully elucidated. In this study, therefore, we investigated the molecular mechanism underlying the anti-inflammatory activity of amentoflavone.

## 2. Materials and Methods

### 2.1. Materials

Amentoflavone (>99% purity), quercetin, phorbol 12-myristate 13-acetate (PMA), 3-4,5-dimethylthiazol-2-yl)-2,5-diphenyltetrazolium bromide (MTT), pam3CSK, and lipopolysaccharide (LPS; *E. coli* 0111:B4) were purchased from Sigma Chemical Co. (St. Louis, MO, USA). Ethyl acetate fraction (Tn-EE-EA) was prepared from 70% ethanol extract of *Torreya nuncifera *leaves (Kyungdong Oriental Medicine Market, Seoul, Republic of Korea; identification by Professor Sukchan Lee (Sungkyunkwan University, Suwon, Republic of Korea); and a voucher specimen number: SKKUMI-0101-Tn-EE-EA), according to general extraction method [18]. U0126, SB203589, and SP600125 were obtained from Calbiochem (La Jolla, CA, USA). Luciferase constructs containing promoters sensitive to CREB and AP-1 were used as reported previously [19, 20]. Enzyme immunoassay (EIA) kits for the determination of PGE_2_ levels were purchased from Amersham (Little Chalfont, Buckinghamshire, UK). Fetal bovine serum and RPMI1640 were obtained from Gibco (Grand Island, NY, USA). The murine macrophage cell line RAW264.7 cells and the human embryonic kidney cell line HEK293 cells were purchased from ATCC (Rockville, MD, USA). All other chemicals were of analytical grade and were obtained from Sigma. Phosphospecific and/or total antibodies to c-Jun, c-Fos, ERK, p38, JNK, I*κ*B*α*, myelin basic protein (MBP), lamin A/C, and *β*-actin were obtained from Cell Signaling (Beverly, MA, USA).

### 2.2. High-Performance Liquid Chromatography (HPLC) Analysis

The content of amentoflavone in Tn-EE-EA was identified by HPLC analysis [21]. The system was equipped with a model K-1001 HPLC pump, model K-2600 fast scanning spectrophotometer, and a model K-500 4-channel degasser (all from KNAUER WellChrom, Berlin, Germany). Elution solvent was buffer A (acetonitrile: 0.5% acetic acid = 40 : 60). A phenomenex Gemini C_18_ ODS (250 × 4.60 mm, 5 *μ*m) column was used as reported previously [22]. 

### 2.3. Cell Culture

RAW264.7 and HEK293 cells were cultured in DMEM or RPMI1640 medium supplemented with 10% heat-inactivated fetal bovine serum (FBS; Gibco, Grand Island, NY, USA), glutamine, and antibiotics (penicillin and streptomycin) at 37°C under 5% CO_2_. For each experiment, cells were detached with a cell scraper. At the cell density used for the experiments (2 × 10^6^ cells/mL), the proportion of dead cells was less than 1%, as measured by trypan blue dye exclusion.

### 2.4. Cell Viability Test

After incubating RAW264.7 and HEK293 cells (1 × 10^6^ cells/mL) for 18 h, amentoflavone (from 0 to 200 *μ*M) was added to the cells, which were then incubated for another 24 h. The cytotoxic effect of amentoflavone was then evaluated using a conventional MTT assay as previously described [23]. At 3 h prior to culture termination, 10 *μ*L of MTT solution (10 mg/mL in phosphate buffered-saline, pH 7.4) was added to each well, and the cells were continuously cultured until the experiment ended. The incubation was halted by the addition of 15% sodium dodecyl sulfate (SDS) to each well, which results in the solubilization of the formazan [24, 25]. Absorbance at 570 nm (OD_570–630_) was measured using a SpectraMax 250 microplate reader (BioTex, Bad Friedrichshall, Germany).

### 2.5. Determination of NO and PGE_2_ Levels

After incubating RAW264.7 cells (1 × 10^6^ cells/mL) for 18 h, the cells were pretreated with amentoflavone (from 0 to 400 *μ*M) for 30 min and then further incubated with LPS (1 *μ*g/mL) or pam3CSK (10 *μ*g/mL) for 24 h. The inhibitory effect of amentoflavone on the production of NO and PGE_2_ was determined by analyzing NO and PGE_2_ levels using Griess reagent and enzyme-linked immunosorbent assay (ELISA) kits, as described previously [26, 27]. 

### 2.6. Semiquantitative Reverse Transcriptase (RT) and Real-Time Polymerase Chain Reaction (PCR) Analysis of mRNA Levels

To determine the mRNA expression levels of various cytokines, total RNA was isolated from LPS-activated RAW264.7 cells using TRIzol Reagent (Gibco BRL), according to the manufacturer's instructions. Briefly, RAW264.7 cells were pretreated with AF (from 0 to 200 *μ*M) for additional 30 min. After that, LPS (1 *μ*g/mL) was exposed to the cells for 6 h. Total RNA was stored at −70°C until use. Semiquantitative RT reactions were conducted as reported previously [28, 29]. The primers used (Bioneer, Daejeon, Republic of Korea) are listed in Table 1.

### 2.7. Luciferase Reporter Assay

HEK293 cells (1 × 10^6^ cells/mL) in a 12-well plate were transfected with 1 *μ*g of plasmid containing CREB-Luc or AP-1-Luc along with *β*-galactosidase using the calcium phosphate method according to the manufacturer's protocol [30]. In case of RAW264.7 cells, the plasmids were transfected to the cells (1 × 10^6^ cells/mL) with Lipofectamine 2000 (Invitrogen, Grand Island, NY, USA) to enhance transfection efficiency of the plasmids. After transfection, cells were further incubated for 24 h. Then, AF was treated to the cells in the presence or absence of PMA (100 nM), TNF-*α* (15 ng/mL), forskolin (2 *μ*M), or LPS (1 *μ*g/mL). 18 h later, the cells were lysed to measure luciferase activities. Luciferase assays were performed using the Luciferase Assay System (Promega) as reported previously [31]. Luciferase activity was normalized to *β*-galactosidase activity, measured at 405 nm, by enzymatic reaction with X-gal and lysate for 5 min at 37°C.

### 2.8. Preparation of Cell Lysates, Nuclear Fractionation, Immunoblotting, and Immunoprecipitation

RAW264.7 cells (5 × 10^6^ cells/mL) were washed three times in cold PBS with 1 mM sodium orthovanadate, lysed using a sonicator in lysis buffer (20 mM Tris-HCl, pH 7.4, 2 mM EDTA, 2 mM ethylene glycol tetraacetic acid, 50 mM *β*-glycerophosphate, 1 mM sodium orthovanadate, 1 mM dithiothreitol, 1% Triton X-100, 10% glycerol, 10 *μ*g/mL aprotinin, 10 *μ*g/mL pepstatin, 1 mM benzamidine, and 2 mM phenylmethylsulfonyl fluoride), and then incubated for 30 min with rotation at 4°C. The lysates were clarified by centrifugation at 16,000 ×g for 10 min at 4°C and stored at −20°C until needed.

Nuclear lysates were prepared using a three-step procedure [32]. After treatment, the cells were collected with a rubber policeman, washed with PBS, and lysed in 500 *μ*L of lysis buffer containing 50 mM KCl, 0.5% Nonidet P-40, 25 mM HEPES (pH 7.8), 1 mM phenylmethylsulfonyl fluoride, 10 *μ*g/mL leupeptin, 20 *μ*g/mL aprotinin, and 100 *μ*M 1,4-dithiothreitol (DTT) on ice for 4 min. Cell lysates were then centrifuged at 19,326 ×g for 1 min in a microcentrifuge. In the second step, the nuclear fraction pellet was washed once in washing buffer, which was the same as the lysis buffer without Nonidet P-40. In the final step, nuclei were treated with an extraction buffer containing 500 mM KCl, 10% glycerol, and the other reagents in the lysis buffer. The nuclei/extraction buffer mixture was frozen at −80°C and then thawed on ice and centrifuged at 19,326 ×g for 5 min. The supernatant was collected as a nuclear extract. Soluble cell lysates were immunoblotted, and protein levels were visualized as previously reported [33]. For immunoprecipitation, cell lysates containing equal amounts of protein (500 *μ*g) from RAW264.7 cells (1 × 10^7^ cells/mL) treated with or without LPS (1 *μ*g/mL) for 2.5 min were precleared with 10 *μ*L protein A-coupled Sepharose beads (50% v/v) (Amersham, UK) for 1 h at 4°C. Precleared samples were incubated with 5 *μ*L of anti-c-Fos antibody overnight at 4°C. Immune complexes were mixed with 10 *μ*L protein A-coupled Sepharose beads (50% v/v) and rotated for 3 h at 4°C.

### 2.9. Enzyme Assay

To evaluate the inhibition of the kinase activity of purified ERK, a kinase profiler service from Millipore was used. In a final reaction volume of 25 *μ*L, ERK1 (human), ERK2 (human), or MAPK kinase (MEK) 1 (human) (1–5 mU) was incubated with reaction buffer. The reaction was initiated by the addition of MgATP. After incubating the mixture for 40 min at room temperature, the reaction was stopped by the addition of 5 mL of a 3% phosphoric acid solution. Ten microliters of the reaction product was then spotted onto a P30 Filtermat and washed three times for 5 min in phosphoric acid (75 mM) and once in methanol prior to drying and scintillation counting. To determine the effect of amentoflavone on LPS-activated ERK activity, immunoprecipitated ERK (prepared from RAW264.7 cells (5 × 10^6^ cells/mL) that had been treated with LPS in the presence or absence of amentoflavone) was incubated with MBP according to the manufacturer's instructions. ERK kinase activity was determined using an anti-phospho-MBP antibody after immunoblotting analysis, as reported previously [34].

### 2.10. Statistical Analysis

**Figure 2:**
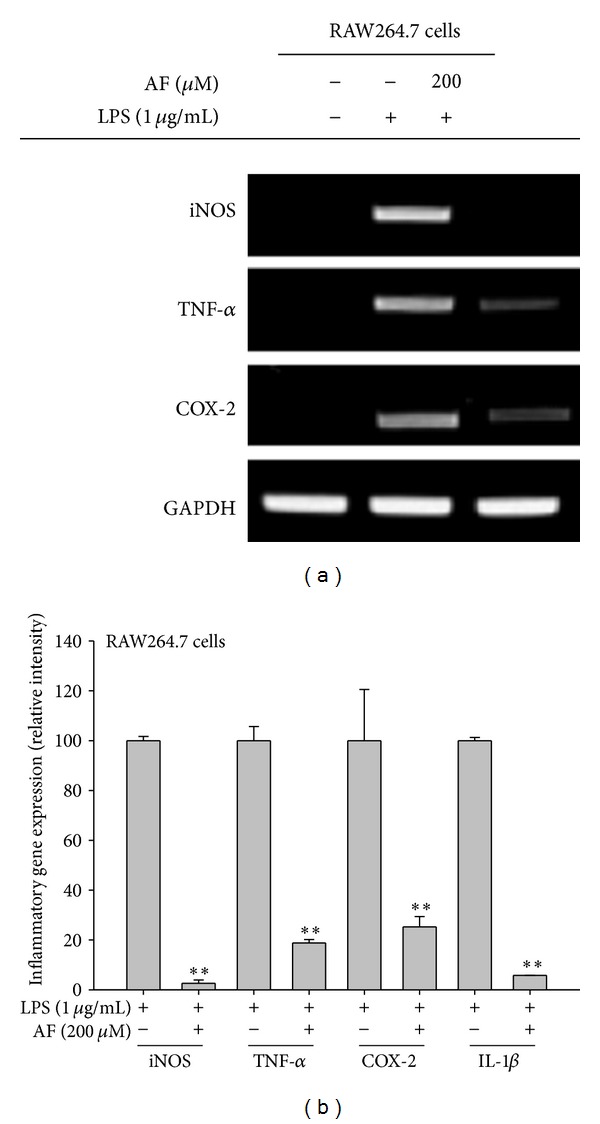
Effect of amentoflavone on mRNA expression of proinflammatory genes. (a and b) The mRNA levels of iNOS, COX-2, IL-1*β*, and TNF-*α* in RAW264.7 cells pretreated with or without AF (200 *μ*M) for 30 min and then exposed to LPS (1 *μ*g/mL) for 6 h were determined using semiquantitative (a) and real-time (b) PCR. RI: relative intensity. **P < 0.01 compared to the control.

**Figure 3:**
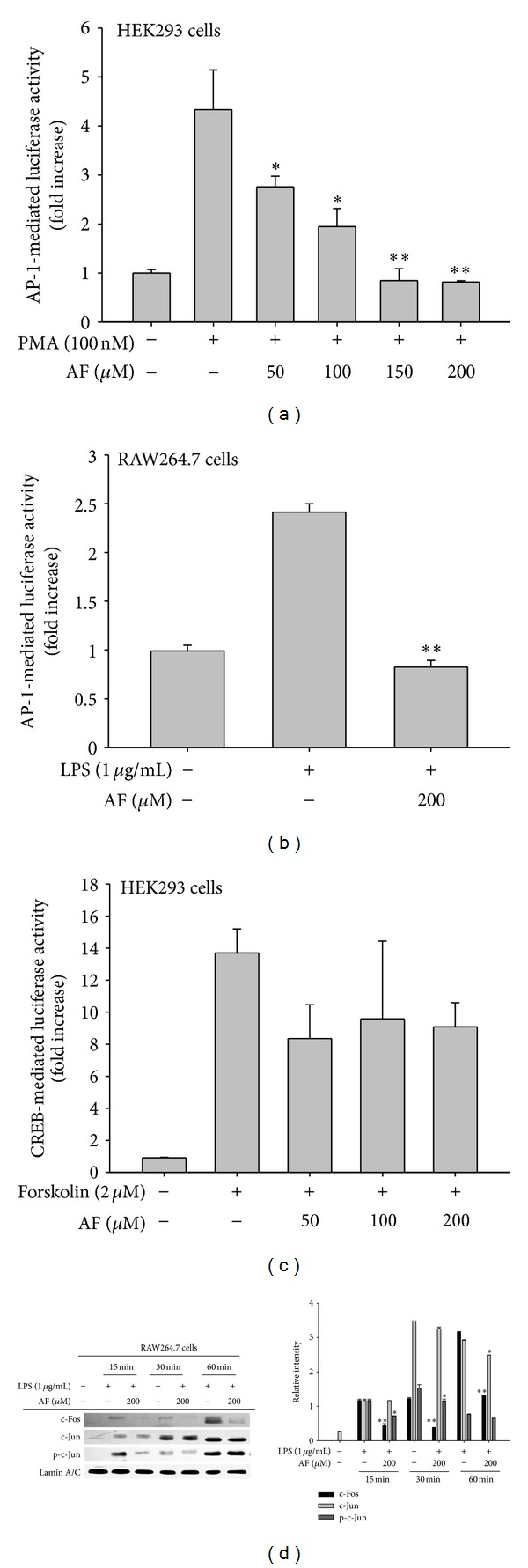
Effect of amentoflavone on transcriptional control of inflammatory genes. (a, b, and c) HEK293 or RAW264.7 cells cotransfected with an AP-1-Luc or CREB-Luc plasmid construct (1 *μ*g/mL each) and *β*-gal (as a transfection control) were treated with amentoflavone (from 0 to 200 *μ*M) in the presence or absence of TNF-*α* (15 ng/mL), PMA (100 nM), forskolin (2 *μ*M or LPS (1 *μ*g/mL). Luciferase activity was measured using a luminometer. ((d), left panel) Total and phosphorylated levels of the AP-1 family proteins c-Jun and c-Fos in the nuclear fractions of LPS-treated RAW264.7 cells were determined by immunoblotting analyses using specific antibodies. Relative intensity (left panel) was calculated by the DNR Bio-Imaging system (a GelQuant software Ver. 2.7). RI: relative intensity. *P < 0.05 and **P < 0.01 compared to the control.

**Figure 4:**
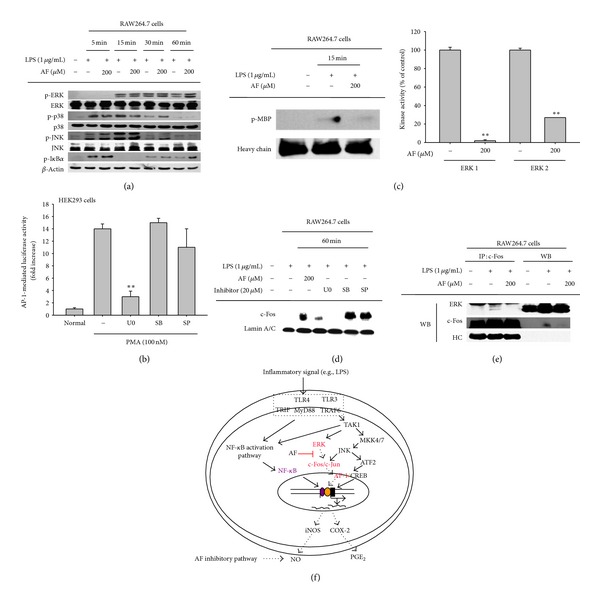
Effect of amentoflavone on signaling factors upstream of AP-1. (a) Phosphorylated and total protein levels of ERK, p38, JNK, and *β*-actin in RAW264.7 cell lysates were determined by immunoblotting analyses using phosphospecific or total protein antibodies. Relative intensities were calculated by densitometric scanning. (b) HEK293 cells cotransfected with an AP-1-Luc plasmid construct (1 *μ*g/mL) and *β*-gal (as a transfection control) were treated with U0126 (U0, 20 *μ*M), SB203580 (SB, 20 *μ*M), or SP600125 (SP, 20 *μ*M) in the presence or absence of PMA (100 nM). Luciferase activity was measured using a luminometer. (c) The kinase activities of immunoprecipitated ERK prepared from LPS-treated RAW264.7 cells and purified ERK (ERK1 and ERK2) were determined in a direct kinase assay using purified enzymes or by measuring the level of phospho-MBP. The control (set as 100%) was the activity of each enzyme (ERK1 or ERK2) obtained after treatment with vehicle. The level of phosphorylated MBP was measured by immunoblotting analysis. (d) The total level of c-Fos in the nuclear fraction of LPS-treated RAW264.7 cells after U0, SB, or SP treatment was determined by immunoblotting analyses using specific antibodies. (e) An interaction between ERK and c-Fos was evaluated by immunoprecipitation and immunoblotting analyses. RAW264.7 cells (5 × 10^6^ cells/mL) were incubated with amentoflavone (200 *μ*M) in the presence or absence of LPS (1 *μ*g/mL) for 30 min. c-Fos was immunoprecipitated from whole cell lysates using a specific antibody, followed by immunoblotting with antibodies to ERK, c-Fos, and rabbit immunoglobulin heavy chain. (f) Putative anti-inflammatory signaling pathway induced by amentoflavone treatment. **P < 0.01 compared to the control.

Data (Figures 1(b), 1(c), 1(d), 1(e), 2(b), 3(a), 3(b), 3(c), 4(b), and 4(c)) are expressed as the means ± standard deviations (SD) calculated from one (*n* = 6) of two independent experiments. Other data are representative of three different experiments with similar results. For statistical comparison, results were analyzed using an analysis of variance/Scheffe's post hoc test and the Kruskal-Wallis/Mann-Whitney tests. All *P* values <0.05 were considered statistically significant. All statistical tests were conducted using SPSS (SPSS Inc., Chicago, IL, USA). 

## 3. Results and Discussion

Amentoflavone is a multipotential biflavonoid compound with antioxidant, anticancer, antiinflammatory, and UV-blocking effects [11–13]. Although the anti-inflammatory activity of amentoflavone has been reported previously [35–37], the molecular targets of this compound have not been fully elucidated. Therefore, in this study, we focused on identifying the molecular target of amentoflavone using LPS-stimulated RAW264.7 macrophage cells. 

Amentoflavone dose dependently suppressed NO (Figures 1(b) left panel and 1(c) left panel) and PGE_2_ (Figures 1(b) right panel and 1(c) right panel) production induced by LPS or pam3CSK in RAW264.7 cells without significantly affecting the viability of RAW264. 7 cells in the absence (Figure 1(d) left panel) or presence (Figure 1(d) left panel) of LPS as well as HEK293 cells (data not shown). Furthermore, this compound alone did not increase NO production (data not shown), indicating that there was no contamination with other immunogens. In particular, a well-known flavonoid compound quercetin also showed significant inhibitory activity on NO and PGE_2_ production Figure 1(g), as reported previously [38], indicating that our experimental systems are well established. The mRNA analysis of iNOS and COX-2 as well as TNF-*α* and IL-1*β*, which are involved in the production of NO and PGE_2_ (Figure 2), demonstrated that amentoflavone-mediated inhibition occurred at the transcriptional level, implying that this compound can block transcriptional activation downstream of LPS-induced TLR4 signaling. Indeed, treatment with amentoflavone dramatically diminished the increase in luciferase activity induced by the activation of AP-1 (Figure 3(a)) but not CREB in HEK293 cells (Figure 3(c)). In agreement with this pattern, upregulated luciferase activity mediated by AP-1 in LPS-treated RAW264.7 cells was also significantly reduced by this compound (Figure 3(b)). Furthermore, amentoflavone treatment strongly decreased c-Fos translocation (from 15 to 60 min) and altered the pattern of c-Jun phosphorylation observed in the nucleus at early time points (15 min) (Figure 3(d)). With previous reports that amentoflavone did inhibit the translocation of NF-*κ*B subunits [35], these data clearly suggest that amentoflavone can also modulate signaling upstream of the translocation of AP-1 (c-Jun and c-Fos). 

Next, therefore, we attempted to identify the target of amentoflavone that mediates its inhibitory role. The major enzymes acting upstream of AP-1 include the MAPKs (ERK, p38, and JNK); therefore, we examined the phosphorylation of these proteins using immunoblotting analysis. As shown in Figure 4(a), there was no inhibition in the phosphorylation of any MAPK upon amentoflavone treatment, indicating that the kinases upstream of ERK, p38, and JNK was not suppressed by this compound. Therefore, we examined whether amentoflavone inhibits any MAPK enzyme directly. To determine which MAPK is relevant for inflammatory signaling in these cells, we first tested the effects of specific inhibitors of ERK, p38, and JNK on AP-1-mediated luciferase activity during PMA stimulation at the concentration (20 *μ*M) with PGE_2_ inhibitory activity (data not shown). Interestingly, the ERK inhibitor U0126, but not the p38 and JNK inhibitors SB203580 and SP600125, strongly suppressed AP-1 activity (Figure 4(b)), implying that ERK regulates PMA-induced AP-1 activation. In fact, PMA has been reported to activate ERK in cells such as neutrophils, monocytic U937, RAW264.7, and Jurkat T cells [39–41]. To test whether amentoflavone can directly suppress the enzyme activity of ERK, we performed a kinase assay using immunoprecipitated ERK and MBP protein as a substrate. As expected, amentoflavone strongly suppressed ERK's kinase activity (Figure 4(c)). Using the same assay, we have previously shown that U0126 strongly inhibits MBP phosphorylation [34]. Here, we also showed that U0126 decreased the translocation of c-Fos (Figure 4(d)). In agreement with these data, amentoflavone disrupted the association between ERK and c-Fos, as assessed by immunoprecipitation and immunoblotting (Figure 4(e)). Interestingly, confocal analysis clearly indicated that LPS treatment induced a condensed localization of ERK in the cytoplasm; under amentoflavone treatment, this pattern was only weakly visible (data not shown). Thus, these results strongly suggest that ERK could be a target of amentoflavone that promotes AP-1 (c-Fos)-mediated inflammatory responses. Since c-Fos is a member of the major AP-1 family [42], AF-mediated inhibition of c-Fos translocation could lead to suppression of AP-1 activity. Moreover, the reduction of c-Jun phosphorylation, an important event for c-Jun binding to DNA [42], could additionally suppress the capacity of c-Jun/DNA binding. Therefore, all of these events could contribute to AF-mediated inhibition of AP-1 activity.

ERK is known to play a pivotal role in inflammatory responses [43]. ERK activity has been clearly shown to be elevated in various inflammatory cells, such as macrophages, monocytic cells, and neutrophils, under inflammatory conditions [44, 45]. In this situation, ERK acts to activate AP-1 and subsequent inflammatory gene expression. For this reason, synthetic and naturally occurring compounds that diminish ERK activity, such as U0126, PD98059, curcumin, resveratrol, and quercetin, have various pharmacological effects in inflammatory diseases [46, 47]. In addition, anti-inflammatory herbal extracts prepared from *Scutellaria baicalensis, Sanguisorba officinalis*, Buyang Huanwu, and Cimicifugae rhizome have been shown to exert their anti-inflammatory effects by targeting ERK [28, 48–50]. Because a synthetic method for mass production of amentoflavone is not yet established, this compound cannot yet be used as a pharmacological agent. Further study of possible mass production methods and their alternatives will be necessary. In this regard, the plant *Torreya nuncifera*, which contains large amounts (1.6%) of amentoflavone (Figure 1(f)) and has been shown to inhibit NO, could be used as an amentoflavone source; here, we show that an extract from *Torreya nuncifera* mimics the effects of amentoflavone on NO production (Figure 1(e)). Therefore, taken together with previous reports, our data strongly suggest that ERK is a prime target of amentoflavone's anti-inflammatory action and that this compound or *Torreya nuncifera *extractscould be used as an anti-inflammatory medication. 

In summary, we have demonstrated that amentoflavone clearly suppresses macrophage-mediated inflammatory responses such as the production of NO and PGE_2_. In particular, this compound strongly inhibited nuclear translocation of c-Fos through the inhibition of its upstream signaling enzyme ERK, as summarized in Figure 4(f). ERK is reported to modulate various inflammatory diseases; therefore, the effects of amentoflavone or *Torreya nuncifera* on inflammatory symptoms will be examined in future experiments.

## Figures and Tables

**Table 1:** Primer sequences used in RT-PCR analysis.

Name		Sequence (5′ to 3′)
Real-time PCR		
iNOS	F	GGAGCCTTTAGACCTCAACAGA
	R	TGAACGAGGAGGGTGGTG
COX-2	F	CACTACATCCTGACCCACTT
	R	ATGCTCCTGCTTGAGTATGT
IL-1*β*	F	GTTGACGGACCCCAAAAAGAT
	R	CCTCATCCTGGAAGGTCCAC
TNF-*α*	F	TGCCTATGTCTCAGCCTCTT
	R	GAGGCCATTTGGGAACTTCT
GAPDH	F	CAATGAATACGGCTACAGCAAC
	R	AGGGAGATGCTCAGTGTTGG
Semiquantitative PCR		
iNOS	F	CCCTTCCGAAGTTTCTGGCAGCAG
	R	GGCTGTCAGAGCCTCGTGGCTTTGG
TNF-*α*	F	TTGACCTCAGCGCTGAGTTG
	R	CCTGTAGCCCACGTCGTAGC
COX-2	F	CACTACATCCTGACCCACTT
	R	ATGCTCCTGCTTGAGTATGT
GAPDH	F	CACTCACGGCAAATTCAACGGCA
	R	GACTCCACGACATACTCAGCAC

## Abbreviations

PGE_2_: Prostaglandin E_2_
NO: Nitric oxide
COX: Cyclooxygenase
iNOS: Inducible NO synthase
TNF-*α*: Tumor necrosis factor-*α*
ERK: Extracellular signal-related kinase
TLR: Toll-like receptors
MAPK: Mitogen-activated protein kinase
AP-1: Activator protein-1
JNK: c-Jun N-terminal kinase
CREB: cAMP response element-binding
EIA: Enzyme immunoassay
ELISA: Enzyme-linked immunosorbent assay
MTT: 3-(4,5-dimethylthiazol-2-yl)-2,5-diphenyltetrazolium bromide
LPS: Lipopolysaccharide
RT-PCR: Reverse transcriptase polymerase chain reaction
MBP: Myelin basic protein.

## References

[1] Ellis AE (2001). Innate host defense mechanisms of fish against viruses and bacteria. *Developmental and Comparative Immunology*.

[2] Harvima IT, Nilsson G (2011). Mast cells as regulators of skin inflammation and immunity. *Acta Dermato-Venereologica*.

[3] Toncic RJ, Lipozencic J, Martinac I, Greguric S (2011). Immunology of allergic contact dermatitis. *Acta Dermatovenerologica Croatica*.

[4] Sekine Y, Yumioka T, Yamamoto T (2006). Modulation of TLR4 signaling by a novel adaptor protein signal-transducing adaptor protein-2 in macrophages. *Journal of Immunology*.

[5] Takeda K, Akira S (2001). Roles of Toll-like receptors in innate immune responses. *Genes to Cells*.

[6] Rüegg C (2006). Leukocytes, inflammation, and angiogenesis in cancer: fatal attractions. *Journal of Leukocyte Biology*.

[7] Ferenčík M, Štvrtinová V, Hulín I, Novák M (2007). Inflammation: a lifelong companion. Attempt at a non-analytical holistic view. *Folia Microbiologica*.

[8] Bharati AC, Sahu AN (2012). Ethnobotany, phytochemistry and pharmacology of Biophytum sensitivum DC. *Pharmacognosy Reviews*.

[9] Baatour O, Mahmoudi H, Tarchoun I (2012). Salt effect on phenolics and antioxidant activities of Tunisian and Canadian sweet marjoram (*Origanum majorana* L.) shoots. *Journal of the Science of Food and Agriculture*.

[10] Ishola IO, Agbaje OE, Narender T, Adeyemi OO, Shukla R (2012). Bioactivity guided isolation of analgesic and anti-inflammatory constituents of *Cnestis ferruginea* Vahl ex DC, (Connaraceae) root. *Journal of Ethnopharmacology*.

[11] Lee C-W, Choi H-J, Kim H-S (2008). Biflavonoids isolated from *Selaginella tamariscina* regulate the expression of matrix metalloproteinase in human skin fibroblasts. *Bioorganic and Medicinal Chemistry*.

[12] Siveen KS, Kuttan G (2011). Effect of amentoflavone, a phenolic component from Biophytum sensitivum, on cell cycling and apoptosis of B16F-10 melanoma cells. *Journal of Environmental Pathology, Toxicology and Oncology*.

[13] Jung SH, Kim BJ, Lee EH, Osborne NN (2010). Isoquercitrin is the most effective antioxidant in the plant *Thuja orientalis* and able to counteract oxidative-induced damage to a transformed cell line (RGC-5 cells). *Neurochemistry International*.

[14] Lee JS, Sul JY, Park JB (2013). Fatty acid synthase inhibition by amentoflavone suppresses HER2/neu (erbB2) oncogene in SKBR3 human breast cancer cells. *Phytotherapy Research*.

[15] Huang N, Rizshsky L, Hauck CC, Nikolau BJ, Murphy PA, Birt DF (2012). The inhibition of lipopolysaccharide-induced macrophage inflammation by 4 compounds in *Hypericum perforatum* extract is partially dependent on the activation of SOCS3. *Phytochemistry*.

[16] Lee SJ, Son RH, Chang HW, Kang SS, Kim HP (1997). Inhibition of arachidonate release from rat peritoneal macrophage by biflavonoids. *Archives of Pharmacal Research*.

[17] Yang JW, Pokharel YR, Kim M-R, Woo E-R, Choi HK, Kang KW (2006). Inhibition of inducible nitric oxide synthase by sumaflavone isolated from *Selaginella tamariscina*. *Journal of Ethnopharmacology*.

[18] Lee YG, Byeon SE, Kim JY (2007). Immunomodulatory effect of *Hibiscus cannabinus* extract on macrophage functions. *Journal of Ethnopharmacology*.

[19] Lee JY, Lee YG, Lee J (2010). Akt Cys-310-targeted inhibition by hydroxylated benzene derivatives is tightly linked to their immunosuppressive effects. *Journal of Biological Chemistry*.

[20] Yu T, Shim J, Yang Y (2012). 3-(4-(tert-Octyl)phenoxy)propane-1,2-diol suppresses inflammatory responses via inhibition of multiple kinases. *Biochemical Pharmacology*.

[21] Aguirre-Hernández E, González-Trujano ME, Martínez AL (2010). HPLC/MS analysis and anxiolytic-like effect of quercetin and kaempferol flavonoids from *Tilia americana* var. mexicana. *Journal of Ethnopharmacology*.

[22] In G, Ahn N-G, Bae B-S, Han S-T, Noh K-B, Kim C-S (2012). New method for simultaneous quantification of 12 ginsenosides in red ginseng powder and extract: in-house method validation. *Journal of Ginseng Research*.

[23] Pauwels R, Balzarini J, Baba M (1988). Rapid and automated tetrazolium-based colorimetric assay for the detection of anti-HIV compounds. *Journal of Virological Methods*.

[24] Shen T, Lee J, Park MH (2011). Ginsenoside Rp1, a ginsenoside derivative, blocks promoter activation of iNOS and Cox-2 genes by suppression of an IKK*β*-mediated NF-*κ*B pathway in HEK293 cells. *Journal of Ginseng Research*.

[25] Vo VA, Lee JW, Chang JE (2012). Avicularin inhibits lipopolysaccharide-induced inflammatory response by suppressing ERK phosphorylation in RAW 264.7. *Macrophages. Biomolecules and Therapeutics*.

[26] Cho JY, Baik KU, Jung JH, Park MH (2000). *In vitro* anti-inflammatory effects of cynaropicrin, a sesquiterpene lactone, from *Saussurea lappa*. *European Journal of Pharmacology*.

[27] Yayeh T, Jung KH, Jeong HY (2012). Korean red ginseng saponin fraction downregulates proinflammatory mediators in LPS stimulated RAW264. 7 cells and protects mice against endotoxic shock. *Journal of Ginseng Research*.

[28] Yu T, Lee YJ, Yang HM (2011). Inhibitory effect of *Sanguisorba officinalis* ethanol extract on NO and PGE2 production is mediated by suppression of NF-*κ*B and AP-1 activation signaling cascade. *Journal of Ethnopharmacology*.

[29] Lee H, Kim J, Lee SY, Park JH, Hwang GS (2012). Processed *Panax ginseng*, Sun ginseng, decreases oxidative damage induced by tert-butyl hydroperoxide via regulation of antioxidant enzyme and anti-apoptotic molecules in HepG2 Cells. *Journal of Ginseng Research*.

[30] Jin M, Park S, Pyo MY (2009). Suppressive effects of T-412, a flavone on interleukin-4 production in T cells. *Biological and Pharmaceutical Bulletin*.

[31] Jung KK, Lee HS, Cho JY (2006). Inhibitory effect of curcumin on nitric oxide production from lipopolysaccharide-activated primary microglia. *Life Sciences*.

[32] Lee JA, Lee MY, Shin IS, Seo CS, Ha H, Shin HK (2012). Anti-inflammatory effects of *Amomum compactum* on RAW 264. 7 cells via induction of heme oxygenase-1. *Archives of Pharmacal Research*.

[33] Jo SK, Hong JY, Park HJ, Lee SK (2012). Anticancer activity of novel daphnane diterpenoids from *Daphne genkwa* through cell-cycle arrest and suppression of Akt/STAT/Src signalings in human lung cancer cells. *Biomolecules and Therapeutics*.

[34] Shen T, Lee J, Lee E, Kim SH, Kim TW, Cho JY (2010). Cafestol, a coffee-specific diterpene, is a novel extracellular signal-regulated kinase inhibitor with AP-1-targeted inhibition of prostaglandin E2 production in lipopolysaccharide-activated macrophages. *Biological and Pharmaceutical Bulletin*.

[35] Woo ER, Lee JY, Cho IJ, Kim SG, Kang KW (2005). Amentoflavone inhibits the induction of nitric oxide synthase by inhibiting NF-*κ*B activation in macrophages. *Pharmacological Research*.

[36] Sosa S, Pace R, Bornancin A (2007). Topical anti-inflammatory activity of extracts and compounds from *Hypericum perforatum* L. *Journal of Pharmacy and Pharmacology*.

[37] Jeong EJ, Seo H, Yang H, Kim J, Sung SH, Kim YC (2012). Anti-inflammatory phenolics isolated from *Juniperus rigida* leaves and twigs in lipopolysaccharide-stimulated RAW264. 7 macrophage cells. *Journal of Enzyme Inhibition and Medicinal Chemistry*.

[38] Yang Y, Yu T, Jang H-J (2012). *In vitro* and *in vivo* anti-inflammatory activities of *Polygonum hydropiper* methanol extract. *Journal of Ethnopharmacology*.

[39] Cho JY, Katz DR, Chain BM (2003). Staurosporine induces rapid homotypic intercellular adhesion of U937 cells via multiple kinase activation. *British Journal of Pharmacology*.

[40] Jhun BS, Lee JY, Oh YT (2006). Inhibition of AMP-activated protein kinase suppresses IL-2 expression through down-regulation of NF-AT and AP-1 activation in Jurkat T cells. *Biochemical and Biophysical Research Communications*.

[41] Chen C-W, Lee ST, Wu WT, Fu W-M, Ho F-M, Lin WW (2003). Signal transduction for inhibition of inducible nitric oxide synthase and cyclooxygenase-2 induction by capsaicin and related analogs in macrophages. *British Journal of Pharmacology*.

[42] Pennypacker KR, Hong J-S, McMillian MK (1994). Pharmacological regulation of AP-1 transcription factor DNA binding activity. *FASEB Journal*.

[43] Ji R-R (2004). Peripheral and central mechanisms of inflammatory pain, with emphasis on MAP kinases. *Current Drug Targets*.

[44] Guha M, Mackman N (2001). LPS induction of gene expression in human monocytes. *Cellular Signalling*.

[45] Galuppo M, Esposito E, Mazzon E (2011). MEK inhibition suppresses the development of lung fibrosis in the bleomycin model. *Naunyn-Schmiedeberg’s Archives of Pharmacology*.

[46] Oh Y-C, Kang O-H, Choi J-G (2009). Anti-inflammatory effect of resveratrol by inhibition of IL-8 production in LPS-induced THP-1 cells. *The American Journal of Chinese Medicine*.

[47] Hua W-F, Fu Y-S, Liao Y-J (2010). Curcumin induces down-regulation of EZH2 expression through the MAPK pathway in MDA-MB-435 human breast cancer cells. *European Journal of Pharmacology*.

[48] Pan T-L, Wang P-W, Leu Y-L, Wu T-H, Wu T-S (2012). Inhibitory effects of *Scutellaria baicalensis* extract on hepatic stellate cells through inducing G2/M cell cycle arrest and activating ERK-dependent apoptosis via Bax and caspase pathway. *Journal of Ethnopharmacology*.

[49] Chen G, Wu L, Deng C-Q (2011). The effects of BuYang HuanWu Decoction and its effective components on proliferation-related factors and ERK1/2 signal transduction pathway in cultured vascular smooth muscle cells. *Journal of Ethnopharmacology*.

[50] Moon L, Ha YM, Jang HJ (2011). Isoimperatorin, cimiside e and 23-O-acetylshengmanol-3-xyloside from Cimicifugae Rhizome inhibit TNF-*α*-induced VCAM-1 expression in human endothelial cells: involvement of PPAR-*γ* upregulation and PI3K, ERK1/2, and PKC signal pathways. *Journal of Ethnopharmacology*.

